# A newly discovered membrane at the origin of the proximal tendinous complex of the rectus femoris

**DOI:** 10.1007/s00276-022-02954-3

**Published:** 2022-05-10

**Authors:** S. Mechó, I. Iriarte, R. Pruna, R. Pérez-Andrés, A. Rodríguez-Baeza

**Affiliations:** 1 Department of Morphological Sciences (Human Anatomy and Embriology Unit), Faculty of Medicine, Universitat Autònoma de Barcelona, Can Domènech Ave, 08193 Bellaterra (Barcelona), Spain; 2 Department of Radiology, Medical Department of Futbol Club Barcelona, Hospital of Barcelona-SCIAS, Bellaterra (Barcelona), Spain; 3 Department of Physical Medicine and Rehabilitation, Ars Médica Clinics, Bilbao, Spain; 4 Department of Orthopedics and Sports Medicine, ICATME, Barcelona, Spain; 5 Department of Radiology, Gemans Trias i Pujol Hospital, Badalona, Barcelona, Spain; 6 Department of Morphological Sciences (Human Anatomy and Embriology Unit), Faculty of Medicine, Universitat Autònoma de Barcelona, Barcelona, Spain

**Keywords:** Rectus femoris, Proximal rectus femoris tendon, Thigh, Anterior superior iliac spine, Membrane

## Abstract

**Purpose:**

The rectus femoris (RF) forms the anterior portion of the quadriceps muscle group. It has a proximal tendinous complex (PTC) which is constituted by a direct tendon (DT), an indirect tendon (IT), and a variable third head. Direct and indirect tendons finally converge into a common tendon (CT). All the PTC shows a medially sloping in its proximal insertion.We investigated several anatomical specimens and discovered a new component: a membrane connecting the CT with the anterior superior iliac spine. Such membrane constitutes a new origin of the PTC. The aim of this study was to clarify whether this membrane was an anatomical variation of the PTC or a constant structure and to describe its morphology and trajectory.

**Material and methods:**

We dissected 42 cadaveric lower limbs and examined the architecture of the PTC. We paid special attention to the morphology and interaction patterns between the tendons and the membrane.

**Results:**

We demonstrated that the membrane is a constant component of the PTC. It has a lateral to medial trajectory and is in relation to the common tendon, the DT, and IT, which present a medial slope. This suggests that the membrane has an stabilizer role for the PTC, acting as a corrector of the inclined vector of the complex.

**Conclusion:**

The RF injuries are frequent in football. The newly discovered membrane is a constant component of the PTC and its integrity should be included in the algorithm to diagnose injuries.

## Introduction

The rectus femoris (RF) forms the most ventral layer of the quadriceps. It is the only one of the four muscles of the quadriceps complex that crosses two joints. Besides being part of the group of flexor muscles of the hip, it also extends the knee joint, and stabilizes the pelvis in the standing position [6, 14–16, 21]. It is a bipennate muscle composed of fascicles that are pennated obliquely to the central tendon [14]. Its embryological development starts at the 17th stage (crown-rump length 11–14 mm, 41 days post fertilization) according to O’Rahilly et al. [17]. At this stage, we can identify mesenchymal condensations of the femur, tibia, fibula, and premuscle masses. The quadriceps femoris first develops as a single mass overlying the anterior aspect of the middle of the femur’s shaft. Then, at the 20th stage (20 mm embryo) according to O’Rahilly, all different heads of the quadriceps femoris are clearly demarcated and attached to the skeletal apparatus by distinct tendons [18].

**Fig. 1:**
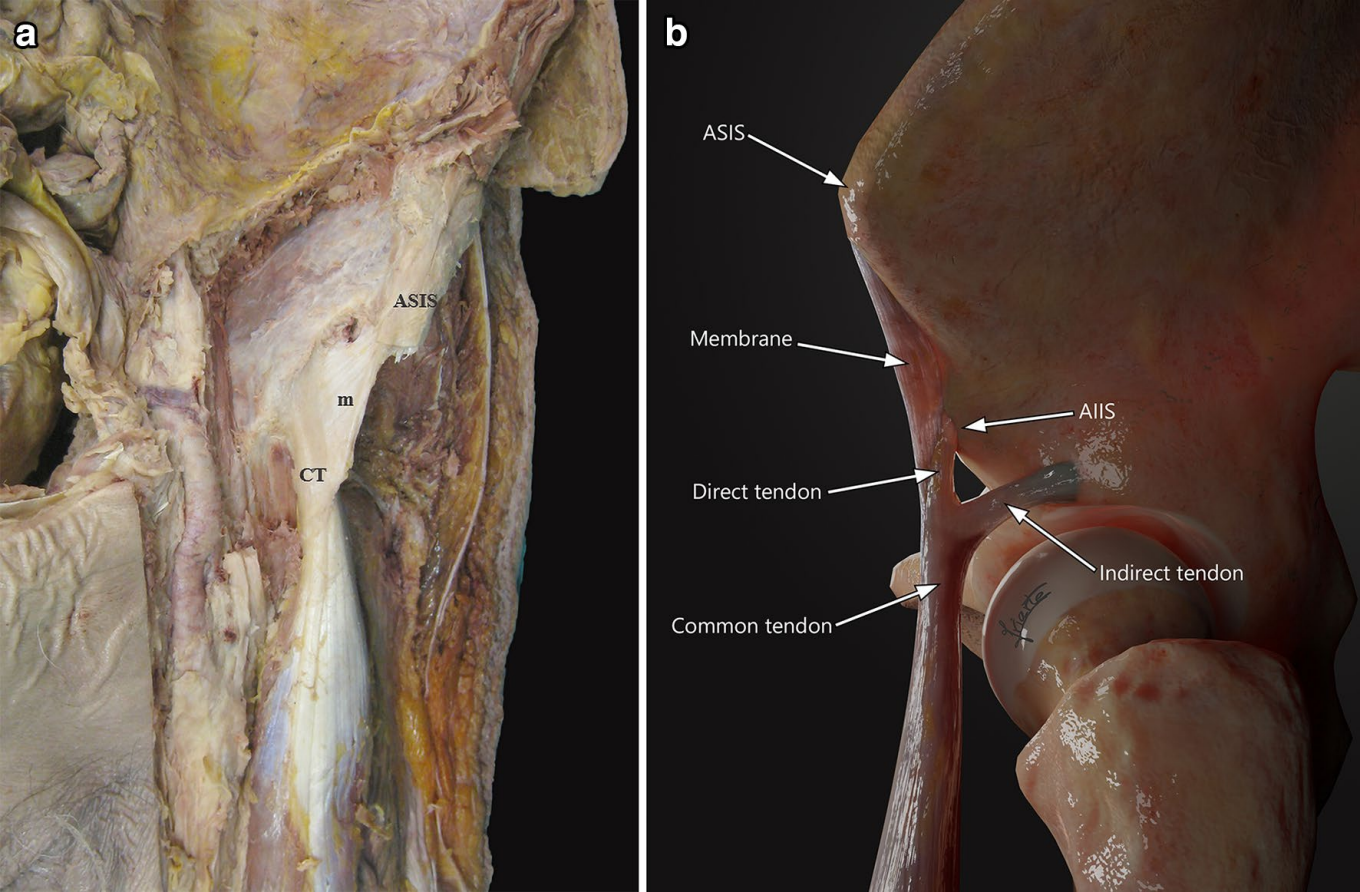
**a** Anterior overview of a left thigh showing the membrane (m) that connects the common tendon (CT) to the anterior superior iliac spine (ASIS). The membrane is opaque. **b** Illustration of a lateral overview of a left thigh showing the proximal tendinous complex (PTC). The membrane is localized anterior to it

Proximal origin of RF is made up of two components, a direct tendon (DT) and an indirect tendon (IT) or reflex (Fig. 1). The IT has been found to develop prior to the DT; indeed, until the sixth fetal month, only the IT can be distinguished [22, 23, 26, 27]. A few centimeters from their origins (approximately 2 cm from the origin of the DT and 5.5 cm from the origin of the IT), both tendons converge in the common tendon (CT), and form the proximal tendinous complex (PTC), a Y-shaped structure covered by a common paratendon [1, 3, 5, 6, 8, 10, 14, 16, 19, 20, 22, 23, 26, 27]. The DT originates from the anterior inferior iliac spine (AIIS) and the underlying rough surface, and it is formed of fibers with a longitudinal craniocaudal direction [1, 6–8, 14, 20, 22]. The IT originates from the supraacetabular sulcus and the lateral aspect of the capsule of the hip joint, and presents fibers in a transverse direction in the axial plane [1, 6–8, 14, 20, 22].

The DT is located more ventrally and presents an inclination that is medial to the longitudinal axis of the muscle (Fig. 1a). It has a short course with a very proximal myotendinous junction in the thigh. Moreover, its fascicular tendinous structure is distributed along the anterior surface of the muscle continuing with the myofascial junction (epimysium, perimysium, and muscle fiber fascicles) [2, 9, 15, 21]. It is mainly involved at the beginning of the hip flexion [3].

The IT has a triangular morphology, follows an anteroposterior course (Fig. 1b), and usually has a medial inclination with respect to the longitudinal axis of the muscle. It extends along the anterior midline of the muscle, forming the central septum. It later thins out and reach the lower third of the thigh, acquiring a linear shape with a long sagittal axis [6, 9, 14, 15]. The myoconnective junction (MCJ) of the IT has a greater craniocaudal extension than the one of the DT [14]. The IT performs its main function as a hip flexor once flexion has begun.

The RF is the component of the quadriceps that is more frequently injured in sports [4]. To better understand all the structures that can be involved in myoconnective injuries, it is necessary to know in detail the anatomic characteristics of the rectus femoris. In this study, we found a membrane-localized anterior to the PTC (Fig. 1a), connecting it to the anterior superior iliac spine (ASIS) that, to our knowledge, has not been previously described.

Hence, our objective is to introduce the newly-discovered membranous structure, determine if it is a fixed component of the PTC, and describe its anatomy and relationship with the PTC.

## Materials and methods

We studied 42 hemipelvis that included the thigh from bodies donated to the Faculty of Medicine of the Universitat Autònoma de Barcelona (UAB). The average age of the subjects was 79 years (range 54–98). 35.72% of the subjects were males and 64.28% females; 50% of the limbs were right and 50% were left.

Body donation at the UAB is regulated by an acceptance document approved by the Ethics Commission in Animal and Human Experimentation (file CEEAH 2904 of March 11, 2015). All hemipelvis were preserved by arterial perfusion of modified Cambridge solution (phenol, ethanol, glycerin, and formaldehyde) and maintained at 6–7°C until their use.

The hemipelvis were dissected and examined using a standardized protocol, by planes from the anterior aspect of the proximal thigh. The skin and subcutaneous cellular tissue were lifted to identify the sartorius muscle (S) and the femoral (Scarpa’s) and quadriceps triangles. The superficial fascia of the S and the vascular and nerve contents of the femoral triangle were also removed to expose the iliopsoas muscle (IP). Afterwards, the IP was dissected from the pelvic brim to its extra-pelvic portion in the proximal thigh, and the inguinal ligament (IL) was sectioned, keeping its insertion in the anterosuperior iliac spine undamaged. The origin of the tensor fasciae latae muscle (TFL) in the quadriceps triangle was identified and the connective tissue anterior to the RF was dissected, between the S and TFL muscles. Then, the S was distally sectioned and moved away respecting its iliac origin, and below, the membrane or fibrous lamina, which is the target of this study appears.

**Fig. 2:**
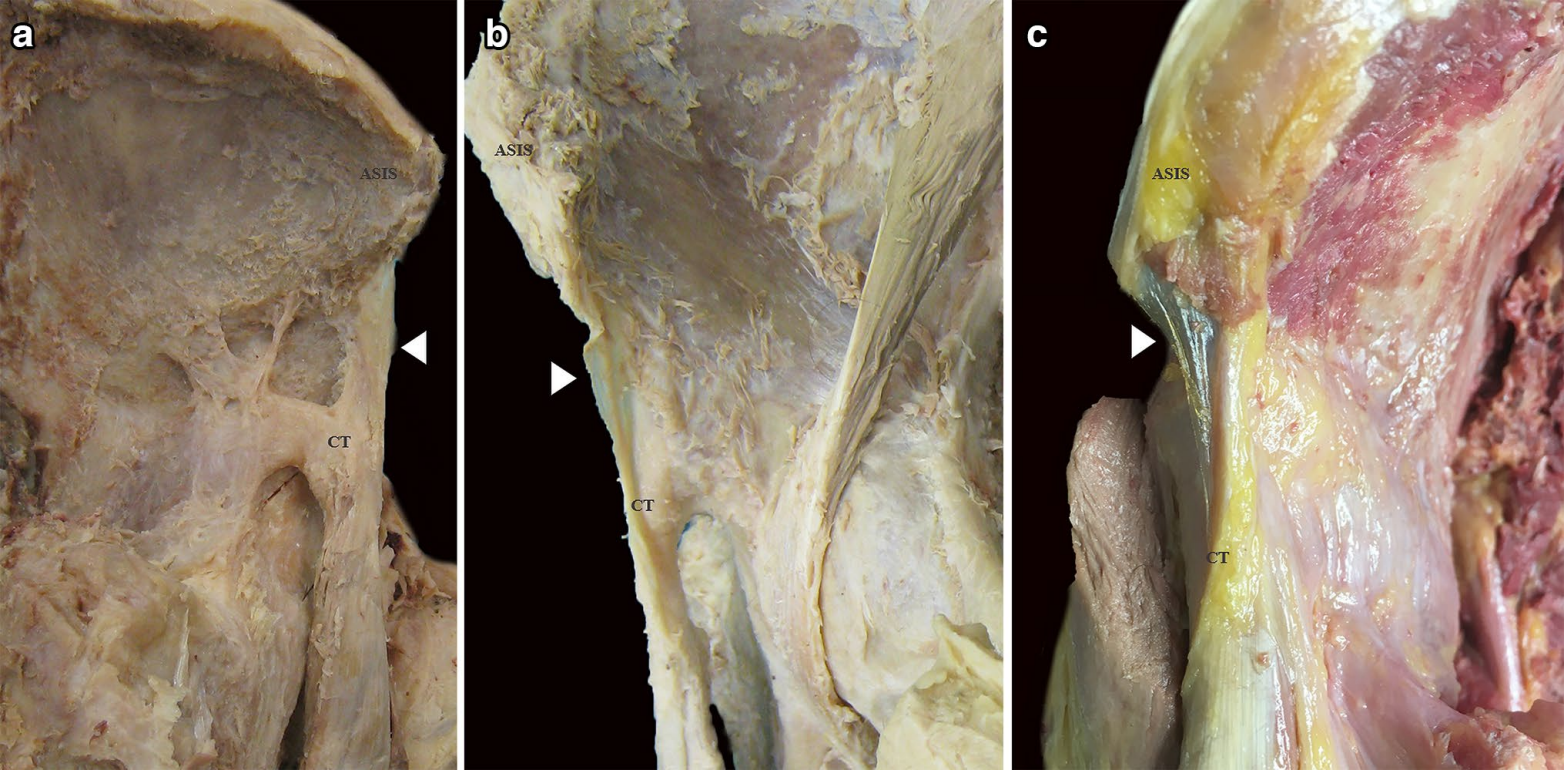
**a** Lateral overview of the PTC of the right rectus femoris muscle (RF). The membrane (arrowhead) shows a deeper anterior margin than the anterior margin of the ASIS. Short and opaque membrane. **b** Lateral overview of the PTC of the left RF. The membrane (arrowhead) shows its anterior margin at the same level than the anterior margin of the ASIS. Medium and transparent membrane. **c** Anterior overview of the PTC of the right RF. The membrane (arrowhead) shows its anterior margin in a more anterior position than the anterior margin of the ASIS. Long and transparent membrane

**Fig. 3:**
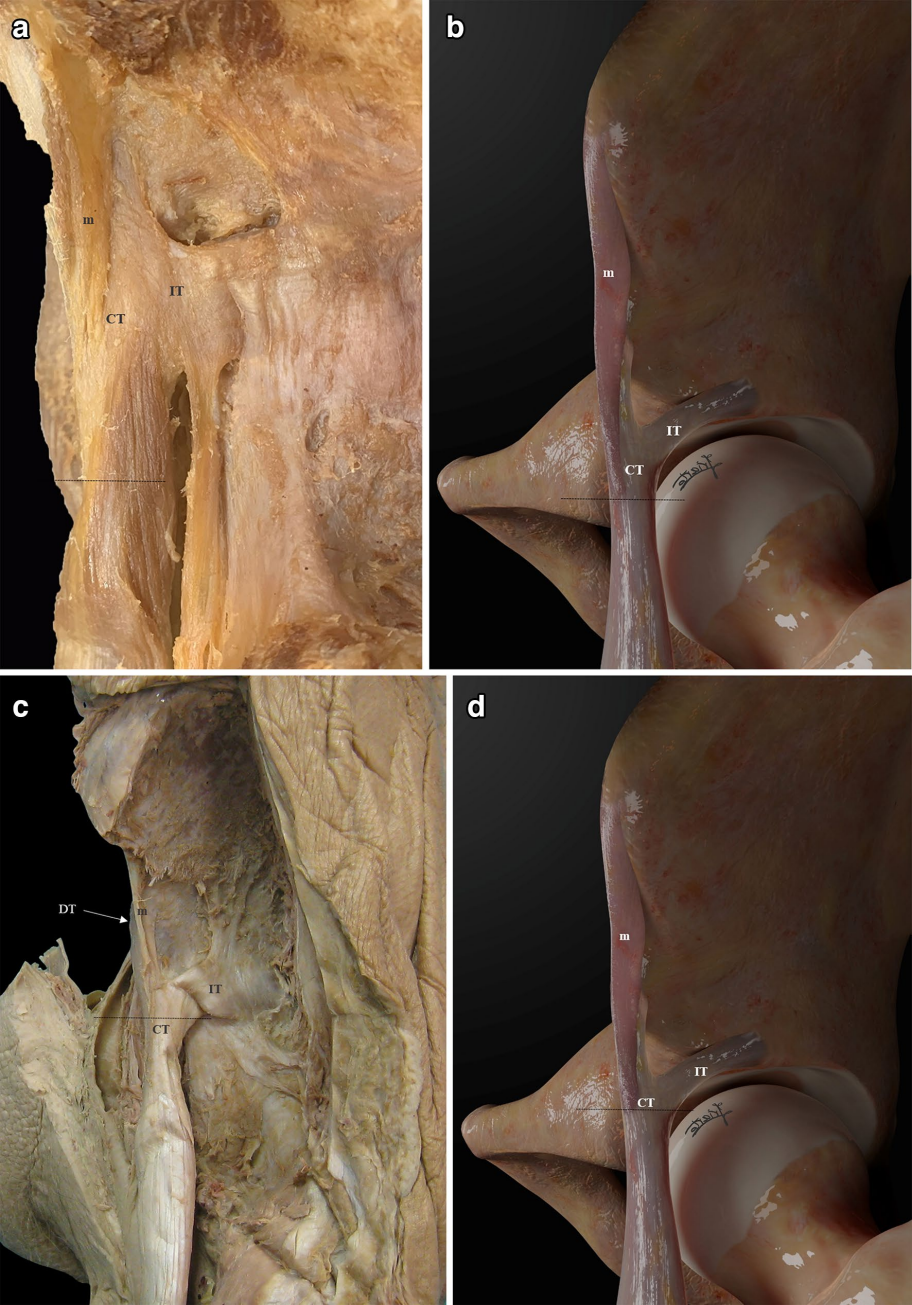
** a** Lateral overview of the PTC of the left RF. The distal margin of the membrane (m) is marked by a dash line at the distal portion of the CT. In this case, we found an independent tendon attached to the IT. **b** Illustration of a lateral overview of the PTC. The distal margin of the membrane (m) is marked by a dash line at the distal portion of the CT. **c** Lateral overview of the PTC of the left RF. The distal margin of the membrane (m) is marked by a dash line at the middle portion of the CT. The anterior aspect of the direct tendon (DT) is behind the membrane. Indirect tendon (IT).** d** Illustration of a lateral overview of the PTC. The distal margin of the membrane (m) is marked by a dash line at the middle portion of the CT

We analyzed different morphological aspects of the membrane: wideness, thickness, and distal insertion in the anteromedial aspect of the CT. The wideness was assessed as the extension in the anteroposterior plane of the membrane with respect to the anterior margin of the ASIS: we considered a membrane short when its anterior margin was deeper than the anterior aspect of the ASIS; and medium or long, when its anterior margin was at the same level or more anterior than the ASIS (Fig. 2). The thickness was assessed according to the opacity of the membrane (opaque or transparent) (Figs. 1a, 2b). Finally, the distal insertion on the anteromedial aspect of the CT was evaluated by dividing the CT into proximal, middle, and distal thirds (Fig. 3).

### Data analysis

The categorical variables were described as absolute frequencies and percentages. The correlations between morphological categories and demographic variables were evaluated using pearson Chi-Square Test. The level of statistical significance was set at *P* < 0.05. The statistical software StatPlus: Mac Pro Version v7 (AnalySoft Inc) was used for data analysis.

## Results

**Fig. 4:**
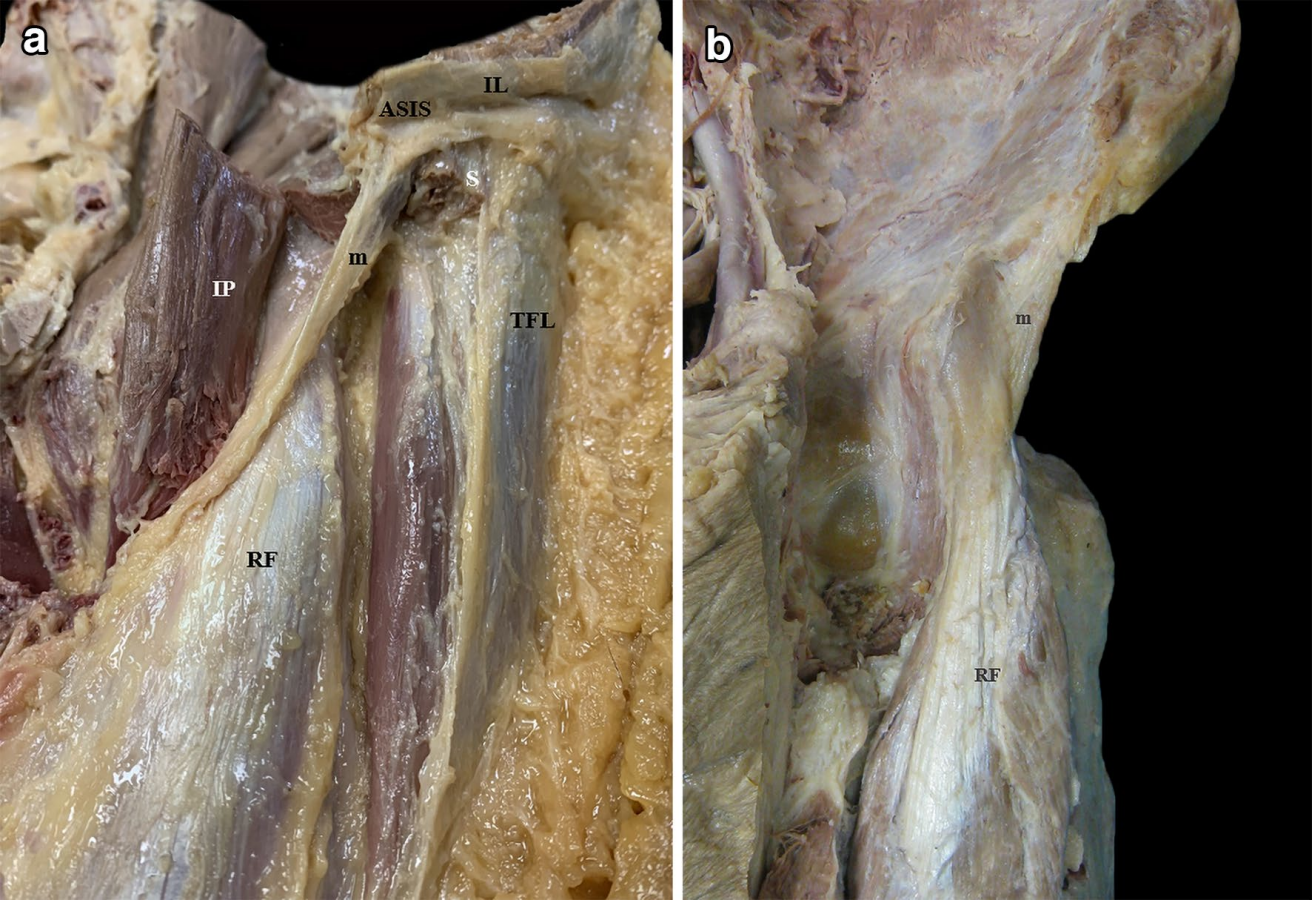
**a** Anterior overview of a left thigh. We can identify the sectioned inguinal ligament (IL) with its insertion in the ASIS. The sartorius muscle (S) was sectioned and removed, the lateral part of the iliopsoas muscle (IP) was sectioned and reclined medially. There is an adipose tissue between the RF and the tensor fasciae latae (TFL) muscle and a longitudinal thickening in the TFL’s fascia. The membrane (m) separates the iliopsoas and sartorius muscles. **b** Anterior overview of the PTC of the left RF. Note the lateral-to-medial-trajectory of the membrane (m) and its medial concavity to accommodate the IP removed

**Fig. 5:**
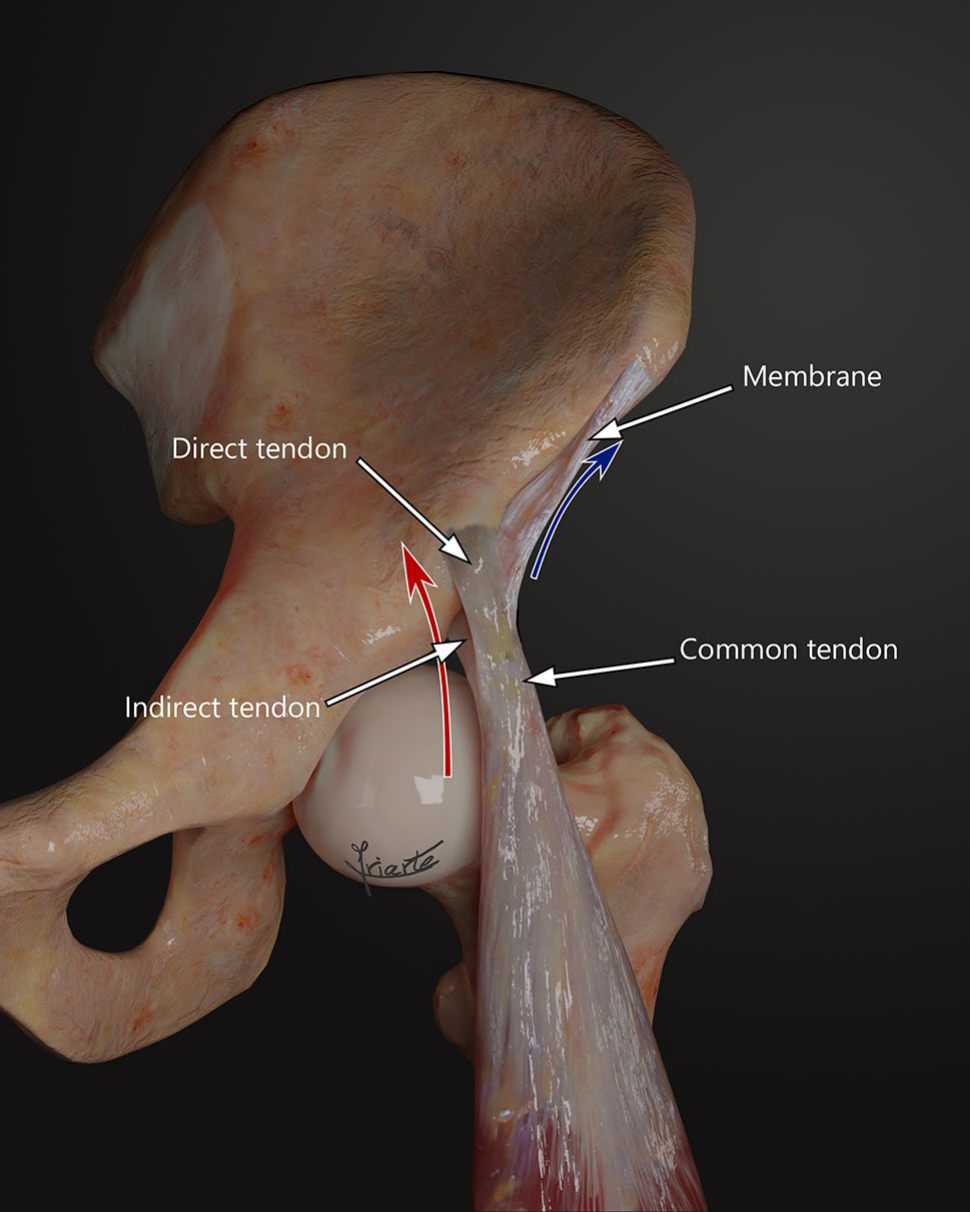
Illustration of an anteromedial overview of a left thigh showing the PTC. The red arrow represents the medial inclination of the PTC with respect to the longitudinal axis of the RF and the blue arrow represents the lateral trajectory of the membrane (colour figure online)

Underneath the S, a fibrous membrane was clearly identified, separating this muscle from the IP (Fig. 4a). The membrane had an oblique course, from lateral to medial, and a medial concavity to accommodate the IP (Fig. 4b). It extended from the distal third (40/42 cases) or the middle third (2/42 cases) of the anterior surface of the CT of the RF to the lower part of the ASIS. It was located deep to the iliac origin of the S and the iliac insertion of the IL. The DT had a medial sloping trajectory to reach its origin in the AIIS; therefore, it was positioned medially to the membrane. In many cases, the IT also had a medial sloping trajectory. Hence, the PTC usually had a medial slope and the membrane a lateral slope (Fig. 5).

**Fig. 6:**
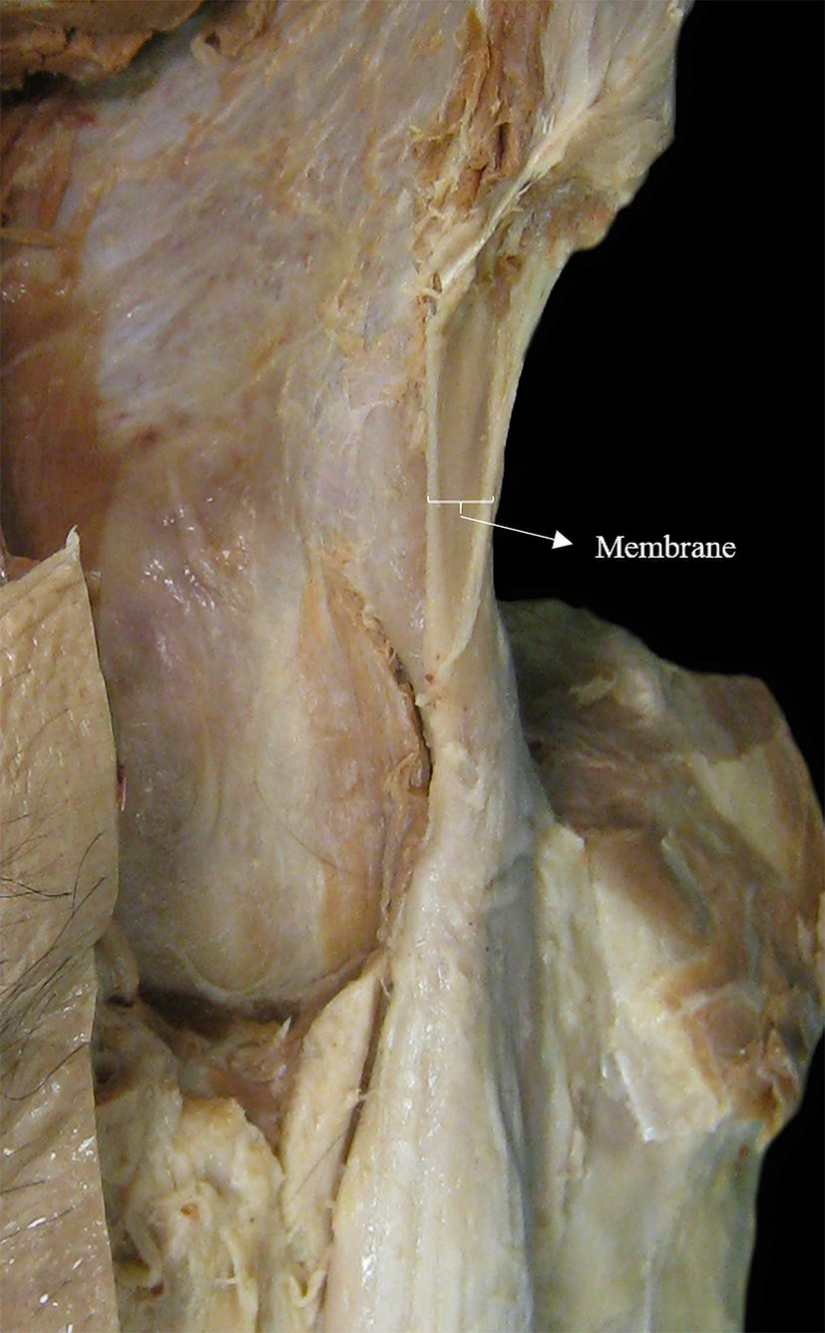
Anterior overview of a left thigh showing a splitting membrane (m)

The membrane was constantly present in all the samples studied. In 23/42 cases it was wide (medium and long membrane), and in 23/42 cases it was slightly thick (opaque). Finally, only in one case presented a canal containing the muscular origin of the S (Fig. 6).

In Table 1, we show the distribution of the different morphological category according to gender and side. In general, there was a greater number of wide (medium and long) membranes and opaque membranes for both genders. However, in the case of the left side, there were more transparent membranes (52.4%). In general, the majority of the membranes show distal insertion in the CT. Finally, there were no significant differences between gender and side (Table 2).

**Table 1:** Morphological characteristics of the membranes according to gender and side. Number of cases in each category (absolute frequencies and percentages)

	Wideness			Thickness		Distal insertion	
	Short	Medium	Large	Transparent	Opaque	Distal	Middle
Female	12 (44.5%)	10 (37%)	5 (18.5%)	12 (44.5%)	15 (55.5%)	25 (92.6%)	2 (7.4%)
Male	7 (46.7%)	6 (40%)	2 (13.3%)	7 (46.7%)	8 (53.3%)	15 (100%)	0
Left	10 (47.6%)	9 (42.9%)	2 (9.5%)	11 (52.4%)	10 (47.6%)	19 (90.5%)	2 (9.5%)
Right	9 (42.9%)	7 (33.3%)	5 (23.8%)	8 (38.1%)	13 (61.9%)	21 (100%)	0

**Table 2:** Statistical results of Pearson Chi-Square test between the different morphological categories, gender and side

	Wideness	Thickness	Distal insertion
Gender	*p* value 0.9	*p* value 0.8	*p* value 0.3
Side	*p* value 0.4	*p* value 0.3	*p* value 0.1

## Discussion

**Fig. 7:**
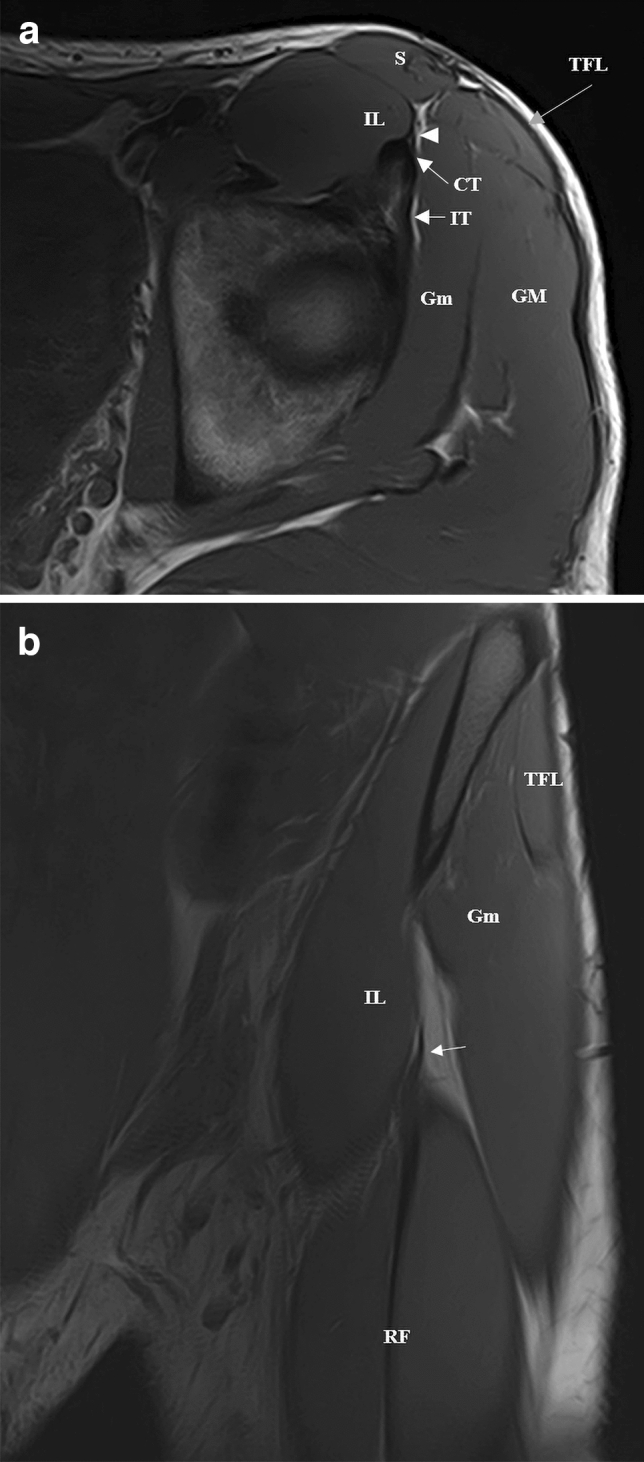
**a** Axial T1 weighted magnetic resonance (MR) image of the left pelvis. We can see the membrane (arrowhead) a thin hypointense band lateral to the iliac muscle (IL) and its relation with the proximal portion of the CT. IT; S; TFL; gluteus minimus (Gm); gluteus magnus (GM). **b** Coronal T1 weighted MR image of the left pelvis. We can see a thicker hypointense membrane (arrow) lateral to the IL and its distal insertion to the distal portion of the CT. Its heterogeneous signal could be secondary to the presence of fat tissue within the connective tissue. RF; S; TFL; Gm

The new membrane that we describe is part of the PTC and is similar to a membrane that has been cited as an anatomical variant of the RF by Macalister in 1875 [13, 25–27]. However, we demonstrated that it is not a variant since it was detected in all the hemipelvis that we studied. It consists of connective tissue belonging to “the fascial system” according to the Fascia Nomenclature Committee [24]. This membrane connects the RF CT to the ASIS, representing an additional origin of the PTC. It is a thin band with a lateral-to-medial trajectory, an inverted triangle shape from distal to proximal, and a variable antero-posterior extension. In this study, we demonstrated that there are no significant differences in the morphological characteristics (thickness, wideness, distal insertion level) of the membrane between gender and side. The membrane thinness, its close relation with the intermuscular fatty planes, and its adjacent position to the epimysium of the IP make it difficult to visualize it by ultrasound. However, using high spatial resolution magnetic resonance imaging (MRI), in the axial and coronal views of the hemipelvis, we can visualize the membrane as a very thin hypointense band in all the sequences (anatomical and fluid sensitive sequences) (Fig. 7).

Muscle lesions are the most common injuries in athletes and they represent more than 30% of the injuries in soccer players [4, 9]. In female professional players, the incidence of muscle injuries is similar to that in males, and the most affected muscle groups are the same (hamstrings, adductors, RF and soleus). However, one study identified in females a predominance of injuries in the quadriceps group (two times more frequent than in men) over the hamstrings [11]. The RF is the component of the quadriceps that is more frequently injured in sports that require repetitive kicking and sprinting, such as soccer and its different variants around the world [14, 15, 22, 28]. The MCJ of the central septum is the most common site of RF injured in soccer. This MCJ depends on the IT of the PTC [14, 15]. Different studies monitored these lesions in Australian footballers and showed that they are associated with a long time to rehabilitate (especially if proximal) and delayed return-to-play [15]. Therefore, from a clinical point of view, we are interested in studying all the elements of the RF and the relationships between them.

We consider the newly discovered membrane as an additional origin of the PTC. Considering the divergence of the DT and the IT, and their medial inclination with respect to the longitudinal axis of the RF, this membrane seems to act as a lateral stabilizer correcting this medial deviation; therefore, it should be included in the radiological algorithm to monitor the PTC of the RF in the presence of a proximal tendon injury. Finally, the assessment of the membrane integrity could help to decide the type of treatment to follow. In cases where both the direct and indirect tendons are injured, Lempainen demonstrated that athletes highly benefit from reattaching the proximal RF [12]. However, in cases of partial injuries, there are different options for the treatment, and it could be important to consider the integrity of the newly discovered membrane within the therapeutic algorithm.

The present anatomical study describes a new component of the PTC of the RF. The PTC shows a medial slope and the membrane has a lateral trajectory to the ASIS; therefore, we could suspect that it has a stabilizing function for the PTC. Future studies could combine these anatomical findings with advanced imaging techniques with two major aims: (1) confirm the possible stabilizing role of the newly discovered PTC membrane; and (2) study the importance to assess the integrity of the membrane to finally choose the most appropriate treatment for proximal RF lesions.

## References

[1] Astzmon R, Sharfman ZT, Atoun E, Sampson TG, Amar E, Rath E (2020). The anatomical properties of the indirect head of the rectus femoris tendon: a cadaveric study with clinical significance for labral reconstruction surgery. Arch Orthop Trauma Surg.

[2] Balius R, Blasi M, Pedret C (2020). A histoarchitectural approach to skeletal muscle injury. the orthopaedic journal of sports medicine.

[3] Bordalo-Rodrigues M, Rosenberg ZS (2005). MR imaging of the proximal rectus femoris musculotendinous unit. Magn Reson Imaging Clin N Am.

[4] Ekstrand J, Hägglund M, Waldén M (2011). Epidemiology of muscle injuries in professional football (soccer). Am J Sports Med.

[5] Gamradt SC, Brophy RH, Barnes R, Warren RF, Byrd JWT, Kelly BT (2009). nonoperative treatment for proximal avulsion of the rectus femoris in professional american football. Am J Sports Med.

[6] Gyftopoulos S, Rosenberg ZS, Schweitzer ME, Bordalo-Rodrigues M (2008). Normal anatomy and strains of the deep musculotendinous junction of the proximal rectus femoris: mri features. Am J Radiol.

[7] Hapa O, Bedi A, Gursan O, Akar MS, Güvencer M, Havitçioglu H, Larson CM (2013). Anatomic Footprint of the Direct Head of the Rectus Femoris Origin: cadaveric study and clinical series of hips after arthroscopic anterior inferior iliac spine/subspine decompression. Arthrosc J Arthrosc Relat Surg.

[8] Hasselman CT, Best TM, Hughes C, Martinez S, Garrett WEJ (1995). An explanation for various rectus femoris strain injuries using previously undescribed muscle architecture. Am J Sports Med.

[9] Isern-Kebschull J, Mechó S, Pruna R, Kassarjian A, Valle X, Yanguas X (2020). Sports-related lower limb muscle injuries: pattern recognition approach and MRI review. Insights Imaging.

[10] Kassarjian A, Rodrigo RM, Santisteban JM (2012). Current concepts in MRI of rectus femoris musculotendinous (myotendinous) and myofascial injuries in elite athletes. Eur J Radiol.

[11] Larruskain J, Lekue JA, Diaz N, Odriozola A, Gil SM (2018). A comparison of injuries in elite male and female football players: A five-season prospective study. Scand J Med Sci Sports.

[12] Lempainen L, Kosola J, Pruna R, Puigdellivol J, Ranne J, Orava S (2018). Operative treatment of proximal rectus femoris injuries in professional soccer player. Orthop J Sport Med.

[13] Macalister A (1875). Additional observations on muscular anomalies in human anatomy. (third series) with a catalogue of the principal muscular variations hitherto published. Trans R Ir Acad.

[14] Mariluis CA, Cupito J, Mamone F (2015). Muscle injuries of the rectus femoris muscle. MR update Revista Argentina de Radiología.

[15] Mendiguchia J, Alentorn-Geli E, Idoate F, Myer GD (2013). Rectus femoris muscle injuries in football: a clinically relevant review of mechanisms of injury, risk factors and preventive strategies. Br J Sports Med.

[16] Moraux A, Wawer R, Lefebvre G, Cotten H, Demondion X, Cotten A (2015). An anatomical study of the indirect tendon of the rectus femoris using ultrasonography. Eur Radiol.

[17] Morelli V, Weaver V (2005) Groin injuries and groin pain in athletes: part 1. Prim Care 32(1):163–83. 10.1016/j.pop.2004.11.011. **PMID: 15831317** 10.1016/j.pop.2004.11.011 15831317

[18] Olewnik L, Tubbs RS, Ruzik K (2021). Quadriceps or multiceps femoris?. Clin Anat.

[19] Ouellette H, Thomas BJ, Nelson E, Torriani M (2006). MR imaging of rectus femoris origin injuries. Skeletal Radiol.

[20] Paturet G (1951). Traité D’Anatomie Humaine. Membres supérieur et inférieur. Tome II. Muscles de la Cuisse.

[21] Peña-Amaro J (2021). The musculotendinous transition of the extracellular matrix. Apunts Sports Med.

[22] Pesquer L, Poussange N, Sonnery-Cottet B (2016). Imaging of the rectus femoris proximal tendinopathies. Skelet Radiol.

[23] Renstrom PA (1992). Tendon and muscle injuries in the groin area. Clin Sports Med.

[24] Schleip R, Hedley G, Yucesoy CA (2019). Fascial nomenclature: update on related consensus process. Clin Anat.

[25] Stringer MD, Kano M, Fausett C, Samalia L (2012). Unilateral short rectus femoris muscle belly. Int J Anat Var (IJAV).

[26] Testut L (1884) Les anomalies musculaires chez l’homme. In:G. Masson (eds) Chapitre II Région antéro-externe de la cuisa. Article II. S1: Variations anatomiques du droit antérieur ou long tríceps. París, Libraire de L'Académie de Médecine. Boulevard Saint-German et rue de l'Éperon. En face de l'École de Médecine, p 608

[27] Tubbs RS, Salter G, Oakes WJ (2004). Femoral head of the rectus femoris muscle. Clin Anat.

[28] Tubbs RS, Stetler J, Savage AJ, Shoja MM (2006). Does a third head of the rectus femoris muscle exist?. Folia Morphol.

